# In vivo ^31^P magnetic resonance spectroscopy study of mouse cerebral NAD content and redox state during neurodevelopment

**DOI:** 10.1038/s41598-020-72492-8

**Published:** 2020-09-24

**Authors:** Radek Skupienski, Kim Q. Do, Lijing Xin

**Affiliations:** 1 Center for Psychiatric Neuroscience, Department of Psychiatry, Lausanne University Hospital (CHUV), Prilly, Switzerland; 2 Center for Biomedical Imaging (CIBM), Ecole Polytechnique Fédérale de Lausanne (EPFL), Lausanne, Switzerland

**Keywords:** Magnetic resonance imaging, Energy metabolism, Neuronal development, Neurophysiology

## Abstract

Nicotinamide adenine dinucleotide (NAD) is an important cofactor of energy-producing pathways. The redox ratio (NAD^+^/NADH) reflects the cellular oxidoreductive state. Oxidative stress and redox dysregulation have been suggested to contribute to various neurological diseases. The assessment of NAD content has been recently demonstrated in large animals and human brains by ^31^P magnetic resonance spectroscopy. However, its measurement in small rodents has never been attempted. The purpose of this study was to investigate, in vivo*,* the NAD content during mouse brain neurodevelopment. ^31^P-MR-spectra were acquired in the mouse brain at postnatal days P20, P40, P90 and P250 at 14.1 T using a 3D-localization sequence. High spectral quality was achieved at 14.1 T. NAD^+^ and NADH were quantified with mean Cramér-Rao lower bound of 10% and 14%, respectively. An increase in NAD^+^/NADH was observed from P20 to P250 due to a decrease in [NADH]. The intracellular pH was significantly reduced with age, while the free [Mg^2+^] in the brain was significantly increased. This study demonstrates for the first time the feasibility of the measurement of NAD content in vivo in mouse brains during development, which opens the prospect of longitudinally studying energy metabolism and redox dysfunction in mouse models of brain pathology.

## Introduction

Nicotinamide adenine dinucleotide (NAD) is a key component of all living cells. NADH (reduced form) and NAD^+^ (oxidized form) are cofactors in bioenergetic pathways, and they play a fundamental role in all oxido-reduction reactions, such as glycolysis, the tricarboxylic acid cycle and the electron transport chain^1,2^. The redox ratio (RR; NAD^+^/NADH) reflects the cellular oxidoreductive state. NAD^+^ is also involved in various other biologically relevant processes, including calcium homeostasis, carcinogenesis, cell death, gene expression and immunological functions^3^.

Physiological changes in redox regulation and oxidative stress, which have been highlighted in developmental and aging processes, are differentially regulated through childhood, adulthood or older age^4^. Pathophysiological oxidative stress and redox dysregulation have been suggested to contribute to many neurological and psychiatric diseases, including schizophrenia, Parkinson’s disease and Alzheimer’s disease^5–7^. An interaction between redox dysregulation and neuroinflammation acting through a vicious circle during brain development has also been described as the center of schizophrenia pathophysiology^8,9^. The fact that NAD could be used as an early biomarker for the detection of neurological conditions that can be routinely assessed appears to be of high importance^10^. The main source of ROS (reactive oxygen species) arises from oxidative energy metabolism in the mitochondria, where a large pool of NAD is used as an electron carrier. Evidence of mitochondrial dysfunction involving NAD and redox ratio anomalies was identified in neurodegenerative conditions^11,12^. Moreover, mitochondrial function is crucial for neurogenesis and neurodevelopment^13^. Redox dysregulation has a direct effect on cellular metabolism and ATP production.

Two ex vivo approaches have generally been used for the assessment of NAD contents: one is based on autofluorescence of the intracellular NADH signal, and the other relies on biochemical analysis. Autofluorescence provides a weak endogenous signal in living cells that is derived mainly from mitochondrial compartments. This approach suffers from low detection sensitivity and limited tissue penetration, with the major drawback being that NAD^+^ is not detected^14^. The biochemical analysis effectuated with HPLC, capillary electrophoresis or enzymatic cycling assays requires a tissue biopsy and some extraction prior to analysis^15–17^. This process might lead to large quantification errors for the highly sensitive redox pairs that have been shown to be rapidly altered after death^18,19^.

The in vivo measurement of NAD^+^ peaks has been demonstrated using ^1^H-magnetic resonance spectroscopy (MRS) from the downfield region of the MR spectra (8.6 to 9.6 ppm); this method does not provide a measurement of NADH, and therefore, similar to the autofluorescence approach, the RR cannot be calculated^20^. The in vivo measurement of both NAD content and redox state has not been available until recently. Thus, there was major interest when ^31^P-MRS, a technique that allows a noninvasive measure of NAD content at high magnetic fields, was demonstrated in cat and human brains^21–25^. The challenge of this measurement by ^31^P-MRS is the intrinsic low sensitivity of ^31^P, together with the fact that the NAD^+^ quartet overlaps the NADH singlet, and both are standing as a shoulder on the right side of the α-ATP resonance signal. Therefore, the high sensitivity and spectral dispersion provided by the high magnetic fields and excellent shimming performance are prerequisites for the in vivo detection of NAD redox pairs. To date, there have been no reports in mice, mainly because the small brain size of a mouse challenges the feasibility of this measurement. However, it is of prime interest to apply this measurement to the mouse brain since they are generally used and subject to genetic modifications for the investigation of pathophysiology. Furthermore, in vivo measurement of these indices during brain development is highly valuable for the investigation of neurodevelopmental pathophysiology. Nevertheless, the measurement in pups is even more challenging due to the smaller size of the brain. Using the ultrahigh field to enhance sensitivity and spectral dispersion would facilitate the measurement of NAD, especially in small volumes.

Therefore, in this study, we first demonstrate the feasibility of the measurement of NAD^+^, NADH and NAD^+^/NADH in vivo during mouse brain development at an ultrahigh magnetic field (14.1 T), where signal sensitivity and spectral dispersion are enhanced. Then, the evolution of the redox state and NAD content during neurodevelopment was investigated by ^31^P-MRS.

We further investigated the phosphoester, intracellular pH (pH^int^) and free magnesium concentrations [Mg^2+^] that can be evaluated by ^31^P-MRS during mouse brain maturation. The different phosphoester metabolites (phosphomonoesters (PME) and phosphodiesters (PDE)) reflect membrane synthesis and degradation and can probe the states of membrane phospholipid metabolism and mitochondrial function^26,27^. The pH^int^ was also determined because of the high dependency between redox reactions and proton concentrations. Mg^2+^ is omnipresent in all living organisms, and regulates energy metabolism as well as mitochondrial function^28^. Mg^2+^ interacts with a variety of molecules, including ATP and is also a known NMDA blocker. Thus, the determination of its concentration will provide complementary comprehension of brain homeostasis, energy metabolism and mitochondrial status^29^.

## Results

### Phantom validation

**Figure 1:**
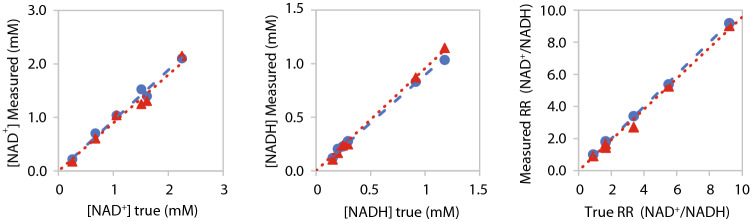
Comparisons between the true values of [NAD^+^], [NADH] and their ratio (RR) in the phantom and those experimentally measured by ^31^P MRS with least square fitting (blue rounds and lines) and the LCModel (red triangles and dots). The linear slope (s) and regression coefficient (R^2^) were 0.94 and 0.98 for NAD^+^ with least square fit and 0.90 and 0.97 with LCModel; 1.12 and 1.00 for NADH with least square fit and 0.96 and 1.00 for NADH with LCModel; 1.12 and 1.00 for RR with least square fit, and 0.99 and 0.98 with LCModel, respectively.

Figure 1 shows the comparison of experimentally measured [NAD^+^], [NADH] and RR with their corresponding true values in the phantoms using two quantification approaches (LCModel and the least square fit). NAD concentrations in phantoms are given in Supplementary Table S1. The linear regression indicates good consistency between the measured and true values for both the least square fit (slope = 0.94–1.12, R^2^ = 0.98–1.00) and LCModel (slope = 0.90–0.99, R^2^ = 0.97–1.00).

### Assessment of quantification methods

**Figure 2:**
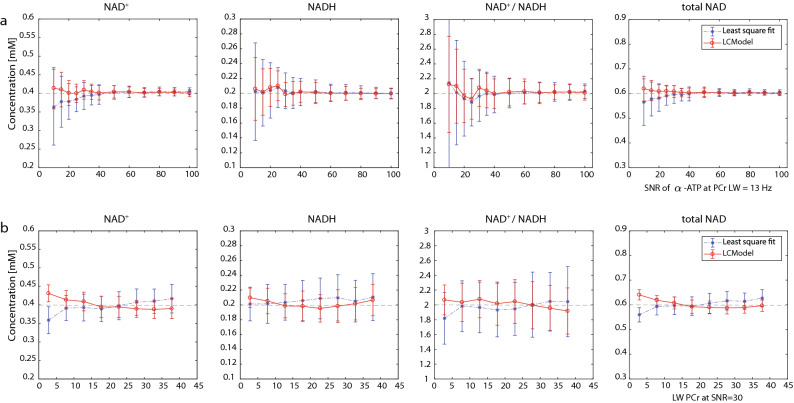
Quantification of [NAD^+^], [NADH], [NAD^+^]/[NADH] ratio (RR) and total NAD ([NAD^+^] + [NADH]) by LCModel or the least square fit at various (**a**) SNR levels and (**b**) linewidths determined with Monte Carlo simulations. LCModel (in red) and least square fit (in blue). The ground truth values are given as dashed lines.

To assess the effect of spectral signal-to-noise ratio (SNR) and linewidth (LW) on the quantification of NAD content, a Monte Carlo simulation was performed. Both the LCModel and least square fit were assessed and compared. Figure 2 shows the means and standard deviations of estimated levels of NAD^+^, NADH, RR and total NAD under different spectral conditions. In general, the LCModel demonstrated a better measurement accuracy over least square fit, especially at SNR < 30, where the least square fit largely underestimated NAD^+^ and total NAD. As expected, the measurement precision reflected by the relative standard deviation (RSD = standard deviation × 100/mean) improved with increases in SNR. The RSD decreased (with SNR 30-100) from 6.1 to 2.4% for the LCModel quantification of NAD^+^ and from 9.2 to 2.3% for the least square fit. The RSD for NADH quantification ranged between 8.1–3.4% and 11.8–3.6%, and for RR quantification, a decrease from 12.1 to 4.9% and 17.4 to 4.8% was observed for LCModel and least square fit, respectively. Finally, the RSD of the total NAD measurement decreased from 4.0 to 1.5% for the LCModel and from 6.0 to 1.7% for the least square fit.

Both quantification methods perform fairly similar in terms of the measurement accuracy at various LWs. The quantification precision improved with narrowing of the LW and such improvement is more pronounced for NADH (from 10.3 to 6.2% for LCModel, from 15.0 to 11.3% for least square fit) and redox ratio (from 16.2 to 9.8% for LCModel, from 23.2 to 19.1% for least square fit) when LW_PCr_ decreases from 38 to 3 Hz (Supplementary Table S3).

### In vivo ^31^P metabolite quantification during brain development

**Figure 3:**
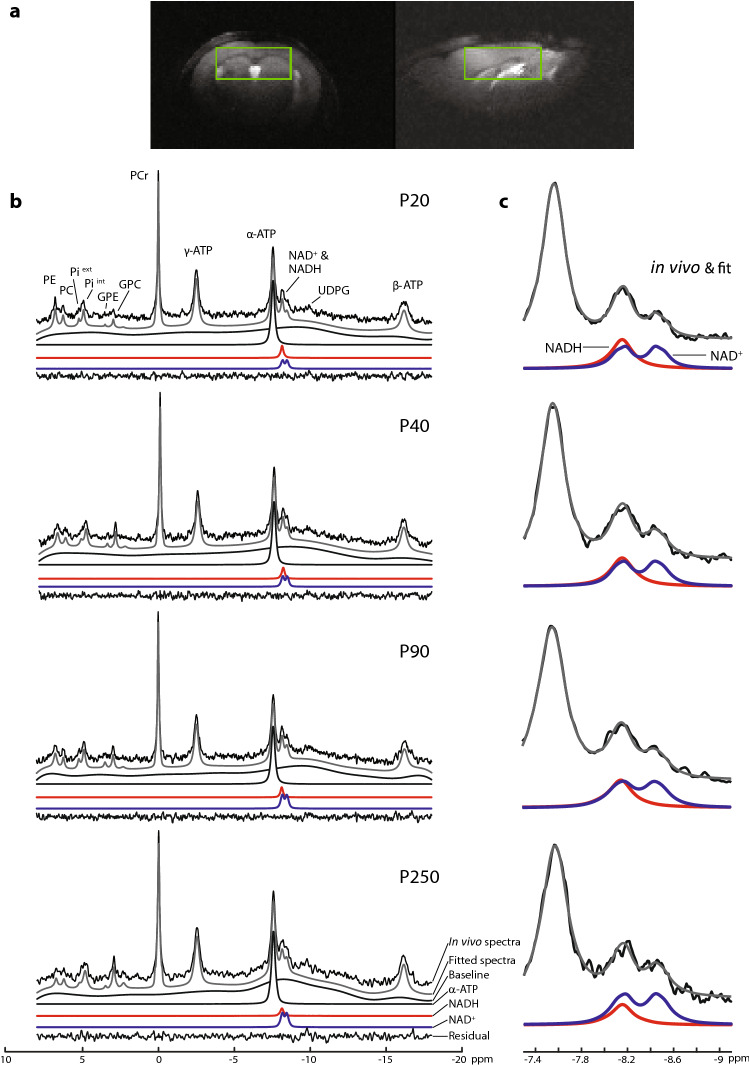
(**a**) VOI location in a female mouse brain at P40 using the fsems images used for positioning. (**b**) Typical in vivo ^31^P MR spectra of the mouse brain from P20 to P250 at 14.1 T (black, no baseline correction, 10 Hz line broadening). The total spectral fit (gray) determined by the LCModel, the baseline, the individual fits of α-ATP, NADH (red), NAD^+^ (blue), and the fitting residual. (**c**) Zoom on the summed spectral region of NAD^+^ and NADH with the following number of animals at a given postnatal days: P20, N = 10; P40, N = 8; P90, N = 8; P250, N = 5. The figure clearly depicts the changes with development in the NAD concentrations.

Typical in vivo ^31^P spectra of the frontodorsal mouse brain obtained from the volume of interest (VOI) depicted in Fig. 3a are shown in Fig. 3b, from P20 to P250. A zoom on the NAD region of the summed spectra in the same age group is shown in Fig. 3c, where the differences in NAD^+^ and NADH levels can be visually observed. All spectra demonstrated excellent sensitivity (SNR = 30–50) and spectral quality (LW_PCr_ = 12–18 Hz) at 14.1 T (Supplementary Table S2), which ensured reliable quantification of the in vivo NAD signals. The respective LCModel fits for NAD^+^, NADH, α-ATP, baseline and fitting residual were demonstrated.

**Figure 4:**
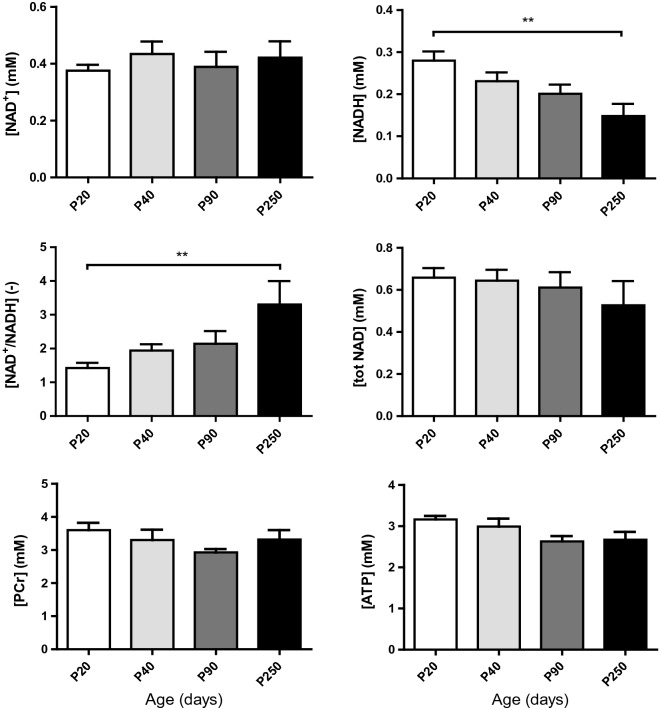
Quantification of brain NAD^+^, NADH, RR, total NAD, PCr and ATP concentrations at postnatal days P20, P40, P90 and P250. Stable NAD^+^ and a decrease in NADH from P20 to P250 led to a strong increase in their ratio, while total NAD was not affected (values are the mean ± SEM); significant differences shown are derived from the post hoc Bonferroni correction test for multiple comparisons, **P < 0.01.

PCr concentrations determined from ^1^H MRS were not significantly different from P20 to P250 (*P* = 0.227, Fig. 4 and Supplementary Table S4). However, ATP levels were shown to be significantly different (*P* = 0.049) and the posttest for linear trend showed a significant decreasing trend with age (*P* = 0.018).

The concentrations of NAD^+^, NADH, NAD^+^/NADH and total NAD (Fig. 4, Supplementary Table S5) were quantified in the mouse brain at P20, P40, P90 and P250 with mean CRLBs of 10% for NAD^+^ and 14% for NADH. For NAD^+^, no significant difference was observed between groups. One way-ANOVA showed a significant difference between age groups in [NADH] (*P* = 0.007 together with a significant linear trend (*P* = 0.0008) of a decrease with age. The RR significantly differed (*P* = 0.006) between age groups and depicted a strong linear trend (*P* = 0.0006) of an increase with age. Bonferroni post hoc tests showed a significant decrease (*P* < 0.01) in NADH levels and a significant increase in the RR (*P* < 0.01) between P20 and P250. The total amount of NAD (tNAD) remained unchanged. With the inclusion of UDPG (uridine diphosphoglucose) in summed spectra analysis, NAD^+^, NADH, RR and tNAD at each age were shown in Supplementary Table S6. From P20 to P250, NADH levels reduced from 0.194 to 0.043 mM, while RR increased from 2.0 to 8.9.

**Figure 5:**
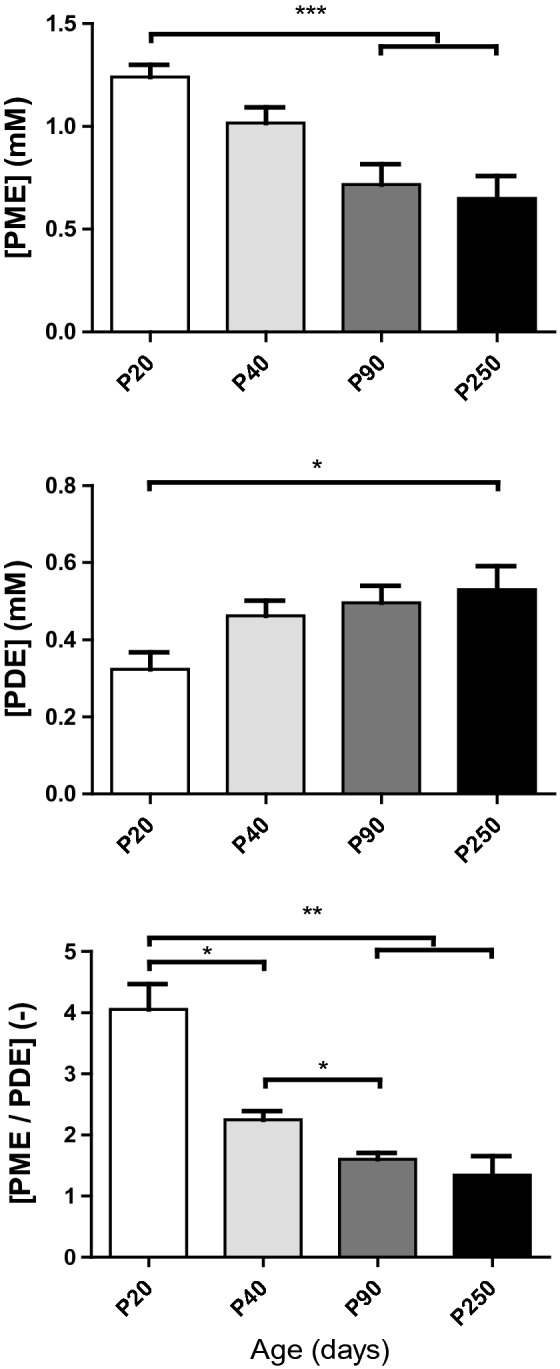
Quantification of brain phosphoesters showing their changes with age. Phosphomonoester (PME) and phosphodiester (PDE) reflect membrane synthesis and degradation, respectively. The PME/PDE ratio reflects decreased membrane turnover from postnatal day P20 to P250 (values are the mean ± SEM); significant differences shown are derived from the post hoc Bonferroni correction test for multiple comparisons *P < 0.05, **P < 0.01, ***P < 0.001.

Phosphoesters are subject to significant changes during development. Among these measured esters (Fig. 5 and Supplementary Fig. S1), PE (*P* = 0.0004) and PME (PC + PE = PME, *P* < 0.0001) significantly decreased with age, while GPC alone (*P* = 0.001) and its sum with GPE (GPC + GPE = PDE, *P* = 0.015) significantly increased with age. A decrease in the monoester with an increase in the diester led to a significant decrease in PME/PDE with age (*P* < 0.0001).

**Figure 6:**
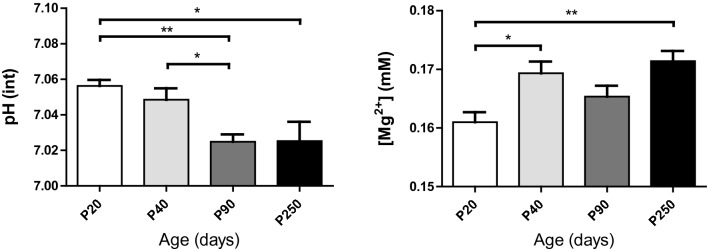
Decrease in the brain intracellular pH and increase in free magnesium concentration with age (values are the mean ± SEM); significant differences shown are derived from the post hoc Bonferroni correction test for multiple comparisons *P < 0.05, **P < 0.01.

The intracellular pH and the free [Mg^2+^] (Fig. 6) were significantly changed with age. A reduction in the pH^int^ (*P* = 0.001) and an increase of the free [Mg^2+^] (*P* = 0.003) were observed in the brain. Bonferroni post hoc tests showed a significant decrease in the pH^int^ (*P* < 0.01) between P20 and P90 as well as between P40 and P90 (*P* < 0.05), while a significant increase in [Mg^2+^] was seen from P20 to P40 (*P* < 0.05) and from P20 to P250 (*P* < 0.01).

### Relationship between NAD content, pH^int^ and [Mg^2+^]

**Figure 7:**
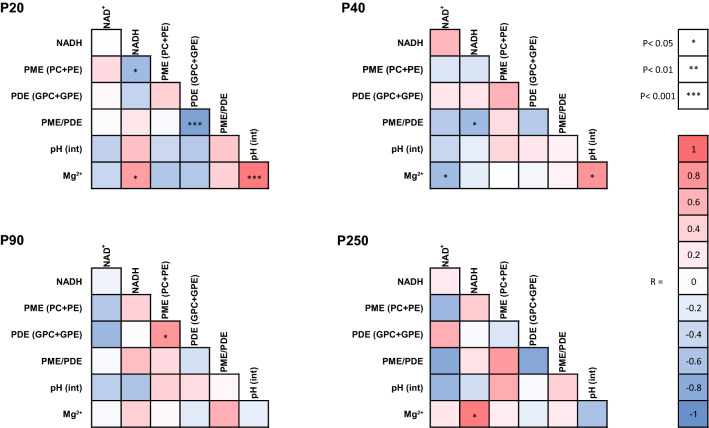
Correlation heat map at postnatal days P20, P40, P90 and P250 of NAD^+^, NADH, phosphoester, pH^int^ and free magnesium [Mg^2+^] content in the mouse brain. The correlation between NADH, and magnesium observed at P20, which disappeared at P40 and P90, reestablished at P250. The strong positive correlation between pH^int^ and magnesium observed at P20 became weaker at P40 and finally resulted in a negative but nonsignificant trend at P250. NADH, which was negatively correlated with PME at P20 and negatively correlated with PME/PDE at P40, appeared to depict a positive but nonsignificant correlation with them at P90 and P250.

At P20, an interconnectivity between pH^int^, [Mg^2+^] and NAD content was present (Fig. 7, Supplementary Fig. S2). Specifically, a strong positive correlation (R^2^ = 0.8646; *P* < 0.0001) was found between [Mg^2+^] and the pH^int^. A positive correlation was found between [Mg^2+^] and NADH (R^2^ = 0.5323; *P* = 0.017). The NAD^+^/NADH ratio was negatively correlated with pH^int^ (R^2^ = 0.5641; *P* = 0.012) and [Mg^2+^] (R^2^ = 0.7522; *P* = 0.001).

pH^int^ remained positively correlated with [Mg^2+^] (R^2^ = 0.6321; *P* = 0.010) at P40, while this correlation disappeared at P90 and tended to be negative at P250. At P250, the positive correlation between NADH and the [Mg^2+^] (R^2^ = 0.8276; *P* = 0.032) was again observed.

## Discussion

This is the first in vivo study demonstrating the measurement of NAD content and redox state during mouse brain development. The high sensitivity and spectral resolution at 14.1 T allowed excellent spectral quality and permitted us to highlight the increase in the RR during development from P20 to P250 together with reductions in PME/PDE and pH^int^ and an increase in [Mg^2+^].

To assess the quantification approaches for NAD content, Monte Carlo simulations were performed using both LCModel and least square fit. For both methods, the measurement precision reflected by the relative standard deviation improved with the increase in SNR and the decrease in spectral linewidth. Therefore, performing measurements at high magnetic fields with excellent shimming performance is beneficial for NAD measurement. At low SNR (< 30) conditions, NAD^+^ can be more accurately quantified by LCModel relative to least square fit (Fig. 2). The large underestimation with least square fit may mainly originate from the worse baseline estimation relative to LCModel at low SNR conditions, as a simulation without inclusion of baseline (data not shown) provided equivalent accuracy with both methods. Note that the measurement precision of NADH was worse than that of NAD^+^, which was because NADH has nearly half of the concentration of NAD^+^, and its peak is closer to the intensive α-ATP resonance, posing additional challenges for its spectral fitting. Taken together, both quantification methods demonstrated rigorous fitting with LCModel having a superior performance at low SNR conditions. Therefore, LCModel was used for subsequent in vivo spectral analyses.

NAD^+^ and NADH are key components involved in many metabolic pathways. This redox couple is responsible for electron transfer and regulates energy metabolism. NADH generated through glycolysis, fatty acid oxidation, and the TCA cycle is an energy-rich molecule that contains a pair of electrons with a high transfer potential. In the electron transport chain, NADH is further oxidized to NAD^+^ with the release of H^+^ and electrons, which continue to drive the electron flow and then reduce O_2_ to H_2_O prior to ATP synthase^30^. Therefore, the RR may serve as a probe for sensing the status of glucose metabolism^22^. In this study, the RR was found to be increased from P20 to P250 in mouse brains, which was driven by a decrease in NADH, suggesting a development towards a higher oxidative brain state from childhood to adulthood^31^. This observation provided the first in vivo evidence supporting adaptive redox regulation with age during neurodevelopment and is in agreement with in vitro results reported with mouse tissue and cultured astrocytes^32^. The reduction of NADH implies developmental changes in glucose metabolism^33,34^. Note that a PET study of human brain development reported that the cerebral metabolic rate of glucose reached a peak at approximately 3–8 years and then declined towards adulthood^35^. For mice, P20 corresponds to 2–3 years of human age^36^. Therefore, the decline in NADH from P20 to P250 may mainly be attributed to the reduction of glucose utilization with maturation. Note that the NAD content and RR measured by ^31^P MRS contain contribution from both cytosolic and mitochondria compartments. Future study by performing compartment-specific manipulation in NAD or RR may facilitate interesting findings of metabolic consequences in different cellular compartments^37^.

Using PCr levels measured from ^1^H-MRS, we observed a mild decrease in ATP, which was often thought to be stable and used as a quantification reference^22,29,38^. This reduction in ATP at adulthood, which is in line with the reduced NADH, suggests that energy homeostasis appears to reach a lower level in the mature brain.

Furthermore, UDPG overlaps with NAD resonances especially that of NADH and has an impact on NAD levels. Other ^31^P-MRS study showed that the inclusion of UDPG in the quantification lowers the NADH level and leads to a higher RR value^24^. Similar results were observed in the current study, with the inclusion of UDPG in the analysis, the NADH values reduced and RR values increased (Supplementary Table S6). The RR values are in good agreement with reported values in 3 and 7 months old mouse brains using HPLC^39,40^. All age-dependent changes in NAD content and RR also remains consistent with those obtained from individual animal analysis, suggesting no impact of the UDPG contribution on current developmental findings.

pH^int^ and magnesium are essential for monitoring and regulating brain physiology.[Mg^2+^] is closely related to ATP synthase, and can also bind to many proteins, including NMDAr^41,42^. Thus, cell energy metabolism and redox state are closely linked to the availability of Mg^2+^. An increase in the pH^int^ together with a decrease in the [Mg^2+^] have been reported upon activation in the human visual cortex^29^. To support a higher energy demand for sustained neuronal activity and maintain ATP homeostasis, increased ATP synthesis is required. This leads to more binding of Mg^2+^ and H^+^ with ADP and ATP, resulting in the reduction of free [Mg^2+^] and [H^+^]. Here, we reveal, in mice, very similar pH^int^ and [Mg^2+^] values from previously described human results^29^. We also highlighted (Fig. 6) a higher pH^int^ together with a lower free [Mg^2+^] in young pups. This suggested that the early developing brain may be in a more energy demanding state than the fully adult brain, mainly for the support of neuronal growth and plasticity together with synaptic proliferation/pruning during brain development^43^.

At P20, pH^int^, [Mg^2+^] and the RR were correlated (Fig. 7, Supplementary Fig. S2), suggesting that during this critical early developmental period, these physiological parameters are tightly regulated. The strong positive correlation between pH^int^ and [Mg^2+^] observed at P20 seemed to disappear at later stages and even tended to be negative at P250, thus reflecting substantial changes in metabolism and the physiological environment with increased age.

In our study, we observed a decrease in PME with an increase in PDE (Fig. 5, Supplementary Fig. S1), which was consistent with the reported results in the rat brain and depicted a marked influence on membrane phospholipid metabolism during brain development^44^. The high PME levels observed in the young developing brain reflect the active synthesis of membranes and are associated with the development of all neural processes. In contrast, PDE levels reflecting more catabolic byproducts were rather low in the young mouse and then steadily rose to their adult levels. The PME/PDE ratio, which reflects membrane phospholipid turnover, was high in the newborn period and rapidly decreased up to P40. Thereafter, the PME/PDE ratio decreases slightly until P250, suggesting that membrane phospholipid breakdown proceeded slightly faster than membrane phospholipid synthesis in the aging process.

The results from age-merged data (Supplementary Fig. S3) showed positive correlations of PME/PDE ratio with NADH and pH^int^. NAD^+^ correlated negatively with pH^int^, and a negative trend was observed between PME and Mg^2+^. Such interconnections among all these components constituted a developmental pattern, suggesting that brain development requires tight and complex cellular regulation. Future studies of neurodevelopmental diseases could monitor this network between NAD, pH, Mg^2+^ and PME/PDE, and search for interruption of this developmental regulatory system and implications of their role in the pathophysiology of diseases.

In conclusion, this study demonstrated for the first time the feasibility of in vivo measurement of NAD^+^, NADH and redox state during mouse brain development by ^31^P-MRS at 14.1 T. This study paves the way to study energy metabolism and redox dysfunction in transgenic mouse models of brain pathologies and aims to develop translational biomarker profiles for early detection of and intervention for neurological and psychiatric diseases. As a noninvasive and nonradioactive strategy, the in vivo measurement of NAD^+^ and NADH by ^31^P-MRS may also serve as a promising biomarker for monitoring brain energy metabolism in future neurodevelopmental studies with children or infants.

## Methods

### Phantom preparation

A set of solutions, depicted in Supplementary Table S1, with known concentrations of NAD^+^ (Roche Diagnostics GMBH, Mannheim, Germany, free acid grade 1, ~ 100%, lot: 11018337) and NADH*3H_2_O (Fluka biochemika, > 97%, lot: 301192 1090), were prepared by dissolving the chemicals in PBS (Sigma life science; D8537, Dulbecco’s phosphate buffered saline, pH 7.1–7.5) containing 9.57 mM inorganic phosphate (Pi), which was used as an internal reference.

### Animal preparation

C57Bl6/j mice born in the local animal facility were housed in ventilated cages on a 12-h light–dark cycle at a room temperature of 20–22 °C with 50–60% humidity. Regular chow and tap water were provided ad libitum.

The experiments were conducted with a cohort of males/females aged 20, 40, 90 and 250 days with body weights of 7–40 g. These ages correspond to different developmental stages: P20 is the end of the suckling period and corresponds to infant stage in human; P40 is the puberty period corresponding to the beginning of adolescence; P90 corresponds to early adulthood stage in human (around 18 years old) and finally P250 corresponds to fully adulthood stage in human (around 30–35 years old)^36,45,46^. The following number of animals were scanned at each age and used for statistical analysis: P20 (5 m/5 f.), P40 (4 m/5 f.), P90 (4 m/4 f.), P250 (4 m/1 f.). For technical reasons such as scanner availability or maintenance, some animals were not scanned at certain time point. As a result, this study is not purely longitudinal.

The animals were anesthetized by a mixture of air:O_2_ (1:1 ratio) and 0.9–1.2% isoflurane. They were then fixed in a mouse holder with a bite bar and two ear inserts (RAPID Biomedical GmbH, Rimpar, Germany). The body temperature, measured with a rectal probe, was kept at 37 ± 0.5 °C by tubing with circulating warm water. Spontaneous breathing was maintained at 90 ± 20 rpm by adjusting the isoflurane concentration. The respiration rate and body temperature were monitored by a small animal monitor (SA Instruments Inc., Stony Brook, NY, USA). All animal procedures were performed according to federal guidelines and were approved by the Swiss cantonal veterinary office.

### ^1^H and ^31^P MR Spectroscopy

All MR experiments were performed on a 14.1 T small animal scanner with a 26 cm horizontal bore (Magnex Scientific, Abingdon, United Kingdom), equipped with a 12 cm internal diameter gradient coil insert (400 mT/m, 120 µs) and a DirectDrive console interface (Agilent Technologies, Palo Alto, CA, USA). Radio frequency transmission/reception was achieved using a homebuilt geometrically decoupled, two single-turn loops (10 mm diameter), quadrature ^1^H surface coil with a linearly polarized one loop ^31^P coil (10 mm diameter).

Fast spin-echo multiple slice images were first acquired in the axial and sagittal directions for voxel positioning using the following parameters: repetition time of 3.3 s, echo time of 43.24 ms, echo train length of 8, interecho spacing of 10.81 ms, field of view of 20 × 20 mm, matrix size of 128 × 128, slice thickness of 0.4 mm, 35 slices and 2 averages. Local shimming in the volume of interest (VOI) was achieved using 1st- and 2nd-order shims with FAST(EST)MAP^47^.

Water suppressed ^1^H-MR spectra were acquired from a volume of 5.76 µL (0.9 × 4 × 1.6 mm^3^) centered in the cerebral cortex using the SPECIAL (SPin ECho full Intensity Acquired Localized spectroscopy) sequence with an echo time of 2.75 ms, a repetition time of 4 s and 240 averages^48^. VAPOR (VAriable Pulse power and Optimized Relaxation delays) water suppression and outer volume suppression were used prior to SPECIAL localization^49^. The transmitter frequency was set on the water resonance to acquire unsuppressed water spectra (8 averages) for metabolite quantification.

^31^P-MR spectra were acquired using a pulse-acquire sequence (adiabatic half passage, 500 µs pulse width, 12 kHz spectral width, 4,096 complex points) in combination with 3D-ISIS to localize the VOI in the frontodorsal part of the brain (Fig. 3a). The following parameters were used: voxel size of 90 µL (2.5 × 6 × 6 mm^3^) at P20 and P40 and 122.5 µL (2.5 × 7 × 7 mm^3^) at P250, TR = 5 s, 1,600 averages (100 blocks of 16 averages, frequency drift and phase variation were corrected prior to the summation of spectra), transmitter offset was set on NAD^+^ (− 8.3 ppm). For phantom experiments: TR = 15 s, 40–100 averages. For scanning NAD peaks and reference Pi signal, the transmitter offset was set on NAD^+^ and on Pi resonances, respectively, due to the asymmetric excitation profile of the adiabatic half passage pulse.

### Monte Carlo simulation

To assess the accuracy and precision of spectral fitting approaches i.e. the least square fit and LCModel (Stephen Provencher Inc., Oakville, Ontario, Canada), Monte Carlo simulations were carried out. ^31^P free induction decay (FID) signals were simulated, including 14 metabolites, with the concentrations and LW (Supplementary Table S2) calculated from the summed in vivo spectra of mouse brains as well as a baseline, also extracted from the summed in vivo signals. To study the effect of the SNR on the quantification of ^31^P metabolites, random Gaussian noise was added to the FID to synthesize 100 ^31^P spectra with an SNR of 10–100 (10/step) using a PCr LW of 13 Hz. Spectra with different LWs were simulated to study the LW effect on the quantification at the experimental SNR (SNR = 30). 100 spectra per point were simulated with an SNR of 30, starting from the LW used in the LCModel basis set (Supplementary Table S2) and increasing it in 7 steps of 5 Hz. The signal-to-noise ratio was defined as SNR = peak height of α-ATP (− 7.6 ppm)/standard deviation of noise level (− 20 to − 25 ppm).

The simulated data were then analyzed by both methods to compare the ^31^P spectral quantification of NAD^+^ and NADH. For the least square fit, ^31^P spectra of NAD^+^, NADH, α-ATP and UDPG with pseudo-Voight spectral shapes were simulated using published chemical shifts and J-coupling constants^21,23,24,50^. These spectra were then used to fit baseline-corrected ^31^P MR spectra using a home written least square error minimization algorithm in MATLAB(R2017a). Briefly, the baseline, originated from short T_2_* components mainly from phospholipids and bones, was removed by subtraction of a spline fit using the backcor() function from MathWorks^51^. Then a region of interest ranging from − 6 to − 14 ppm (PCr was set as 0 ppm) was selected for spectral fitting using the lscurvefit() function from MATLAB.

For LCModel fit, a basis-set was prepared using simulated ^31^P spectra including PCr (phosphocreatine), α − ATP, β − ATP, γ − ATP, Pi^int^ (intracellular inorganic phosphate), Pi^ext^ (extracellular inorganic phosphate), PE (phophothanolamine), PC (phosphocholine), GPC (glycerophosphocholine), GPE (glycerophosphoethanolamine), MP (membrane phospholipid), UDPG, NADH, and NAD^+^, with respective linewidths (Supplementary Table S2)^25,52^.

### Spectral quantification

In vivo ^1^H MR spectra were fitted by LCModel with a basis set containing a measured macromolecules spectrum^53^ and simulated metabolites spectra^54^ to determine metabolite concentrations. Unsuppressed water spectra were used as the quantification reference. The brain water content was measured at P20, P40, and P90 using the weight difference between freshly removed and fast dissected cerebral cortex and its residue after lyophilization (Supplementary Table S4). The water content at P250 was set as that at P90 assuming stable water content at adulthood. The respective water content was incorporated in the control file for metabolite quantification.

Apodization with a 10 Hz exponential function was applied to all ^31^P spectra prior to spectral quantification. In vivo ^31^P MR spectra were analyzed by LCModel using the basis-set described above. For individual animal analysis, UDPG was excluded due to its low SNR. To evaluate the impact of UDPG on the quantification of NAD content, ^31^P MR spectra at each age were summed and quantified with the inclusion of UDPG (Supplementary Table S6). The ^31^P metabolite levels at each age were normalized using the mean PCr level obtained from the ^1^H experiment at the respective age (Supplementary Table S4). T_1_ saturation effect for metabolites with long T_1_s (such as phosphoester) was not corrected assuming the same saturation effect for each age group. The Cramér-Rao lower bound (CRLB) was used as an exclusion criterion, and the cutoff was set at a maximum of 30%. ATP level was reported as the mean of α-ATP and γ-ATP values^55^.

The pH^int^ and free [Mg^2+^] were calculated from specific chemical shift differences between metabolites. Intracellular pH^int^ was determined by Eq. 1 from the chemical shift difference between Pi^int^ and PCr. The parameter δ_Pi_ was the chemical shift difference between PCr and Pi, and the constants used were pKa = 6.73, δ_a_ = 3.275, and δ_b_ = 5.685^56^.

1 $$pH=pKa+{log}_{10}\frac{{\delta }_{Pi}-{\delta }_{a}}{{\delta }_{b}-{\delta }_{Pi}}$$

The free [Mg^2+^] concentration was calculated using the chemical shift difference between PCr and β-ATP (δ_βATP-PCr_) according to Eqs. 2 and 3^41^.

2 $${pMg}^{2+}=4.24-{log}_{10}\left[\frac{{({\delta }_{\beta ATP-PCr}+18.58)}^{0.42}}{{(-15.74-{\delta }_{\beta ATP-PCr})}^{0.84}}\right]$$

3 $$\left[{Mg}^{2+}\right]={10}^{-p{Mg}^{2+}}$$

### Statistical analysis

All analyses were performed in R (R version 3.6.0, 2019, https://cran.r-project.org/), GraphPad Prism 5 (GraphPad software, Inc.) or MATLAB (R2017a). All variables were tested by one-way ANOVA using age as a fixed factor. In the case of significant differences between groups, the effect of age was post hoc investigated between all pairs of columns using the Bonferroni correction for multiple comparisons. A posttest for linear trend was also effectuated followed by a linear regression along age to evaluate whether a small regular increase or decrease with age that would not be detected by ANOVA could be present. A linear mixed effect analysis was effectuated to evaluate the effect of sex, the duration of anesthesia and the time period of the day which did not revealed significant influence of these parameters on our cohort. The results are presented as the mean ± standard error of the mean unless otherwise stated. When exact p-values are not provided, significant differences (*) are considered for *P* < 0.05, very significant differences (**) for *P* < 0.01 and extremely significant differences (***) for *P* < 0.001.

## Supplementary information

Supplementary Information.

## Data Availability

The data that support the plots within this paper and other findings of this study are available from the corresponding author upon reasonable request.
