# Supplementary material for: In vivo 31P magnetic resonance spectroscopy study of mouse cerebral NAD content and redox state during neurodevelopment
Source: Sci Rep. 2020 Sep 24;10:15623. doi: 10.1038/s41598-020-72492-8 (PMC7519085; doi:10.1038/s41598-020-72492-8)
Supplement: Supplementary file 1 — Supplementary Information. [file 41598_2020_72492_MOESM1_ESM.pdf]

Supporting Information

In vivo ^{31}P magnetic resonance spectroscopy study of mouse cerebral NAD content and redox state during neurodevelopment

Radek Skupienski[1,2], Kim Q Do[1] and Lijing Xin[2]

[1]*Center for Psychiatric Neuroscience, Department of Psychiatry, Lausanne University Hospital (CHUV), Prilly, Switzerland,*
[2]*Center for Biomedical Imaging (CIBM), Ecole Polytechnique Fédérale de Lausanne, Lausanne, Switzerland,*

Table S1: Concentration [mM] of NAD^+, NADH and their ratio used in different phantoms for method validation. Inorganic phosphate (Pi) concentration of 9.57 mM was used as an internal reference for all phantom quantification.

$[NAD^+]$	[NADH]	$[NAD^+]$/ [NADH]
1.055	1.180	0.89
1.507	0.914	1.65
0.251	0.152	1.65
0.670	0.199	3.37
1.612	0.293	5.50
2.249	0.244	9.22

Table S2: List of metabolite concentrations and linewidths (LW) used in the Monte-Carlo simulation, metabolite LWs extracted from summed *in vivo* spectra with 10 Hz Lorentzian apodization, and the LWs used for the basis set in LCModel.

Metabolite	*In vivo* Conc. (mM)	*In vivo* LW(Hz)	Monte-Carlo LW(Hz)	Basis set LW(Hz)
α-ATP	2.8	32	19-54	19.3
β-ATP	2.8	60	47-82	47.0
γ-ATP	2.8	37	24-59	23.9
PCr	2.9	16	3-38	2.8
MP	0.07	ND	40-75	40.0
Pi^{int}	0.54	13.5	0.5-35.5	0.5
Pi^{ext}	0.057	20	7-42	6.9
PC	0.25	22	9-44	8.6
PE	0.58	23	10-45	9.9
GPC	0.25	17	4-39	4.3
GPE	0.44	10	3-38	3.3
NAD^+	0.40	30	17-52	16.9
NADH	0.20	30	17-52	16.9
UDPG	0.10	14	1-36	1.0

Table S3: Relative standard deviations (RSD, %) obtained from the Monte Carlo simulation data quantified by LCModel and least square fit (Lsfit) at different SNR and LW.

SNR	RSD (%)							
	NAD^+		NADH		RR		tot NAD	
	LCModel	Lsfit	LCModel	Lsfit	LCModel	Lsfit	LCModel	Lsfit
10	13.5	28.1	20.5	32.5	30.6	74.5	8.1	16.7
15	11.4	18.2	15.5	22.3	23.8	35.1	6.7	11.9
20	8.4	12.7	12.3	18.3	18.3	26.1	5.0	9.3
25	6.2	8.7	9.5	12.9	14.2	17.0	3.7	6.6
30	6.1	9.2	8.1	11.8	12.1	17.4	4.0	6.0
35	5.1	6.6	7.9	10.4	11.4	14.6	2.9	4.2
40	4.8	6.6	7.0	9.1	9.9	12.4	3.1	4.7
50	4.2	4.5	6.7	7.9	9.3	9.8	2.8	3.6
60	3.8	3.6	5.4	5.6	8.4	7.4	2.1	2.7
70	2.9	3.1	4.7	5.2	6.6	7.3	1.8	2.0
80	2.4	2.9	3.5	4.6	4.9	6.2	1.6	2.1
90	2.2	2.8	3.4	3.7	4.9	5.4	1.4	1.8
100	2.4	2.3	3.4	3.6	4.9	4.8	1.5	1.7

LW_{PCr} (Hz)	RSD (%)							
	NAD^+		NADH		RR		tot NAD	
	LCModel	LSfit	LCModel	LSfit	LCModel	LSfit	LCModel	LSfit
2.8	5.3	10.1	6.2	11.3	9.8	19.1	3.2	5.2
7.8	6.0	8.6	8.4	13.0	12.7	17.3	3.2	5.7
12.8	6.1	9.2	8.1	11.8	12.1	17.4	4.0	6.0
17.8	7.2	8.8	10.4	13.2	14.4	18.8	4.6	6.3
22.8	7.2	9.5	9.4	13.2	14.5	18.4	4.4	6.6
27.8	5.9	8.8	11.2	14.9	15.9	21.9	4.2	6.1
32.8	6.5	8.0	10.9	13.7	15.4	19.1	3.9	5.4
37.8	7.0	9.2	10.3	15.0	16.2	23.2	3.9	5.5

Table S4: PCr levels determined from ^{1}H MR measurement and cerebral cortex water concentrations in mouse brains at different ages. All values are in mean ± standard deviation.

Age (days)	[PCr] (mM)	% Water (w/w)
P20	3.603 0.728	78.9 0.42
P40	3.300 0.892	77.7 0.41
P90	2.926 0.289	77.1 0.39
P250	3.319 0.563	77.1*

*assuming water content remains stable at adulthood

Table S5: Levels and CRLBs of NAD^+, NADH, RR and total NAD obtained at P20, P40, P90 and P250, respectively. All values are in mean ± standard deviation.

Age (days)	[NAD^+]		[NADH]		RR	[tNAD]
	Conc. (mM)	CRLB (%)	Conc. (mM)	CRLB (%)	(-)	Conc. (mM)
P20	0.376 0.065	10 ± 1	0.280 0.070	11 ± 2	1.427 0.473	0.656 0.098
P40	0.434 0.133	9 ± 2	0.231 0.064	12 ± 3	1.941 0.564	0.665 0.178
P90	0.389 0.149	10 ± 3	0.201 0.062	14 ± 3	2.145 1.062	0.590 0.156
P250	0.421 0.128	9 ± 1	0.148 0.065	19 ± 5	3.305 1.552	0.569 0.155

Table S6: Levels of UDPG, NAD^+, NADH, RR and total NAD obtained with inclusion of UDPG in the quantification from summed spectra at P20, P40, P90 and P250. All concentration levels are in mM.

Age (days)	[UDPG]	[NAD^+]	[NADH]	RR	[tNAD]
P20	0.147	0.352	0.194	2.033	0.545
P40	0.185	0.368	0.155	2.422	0.523
P90	0.125	0.360	0.108	3.185	0.468
P250	0.206	0.408	0.043	8.880	0.451

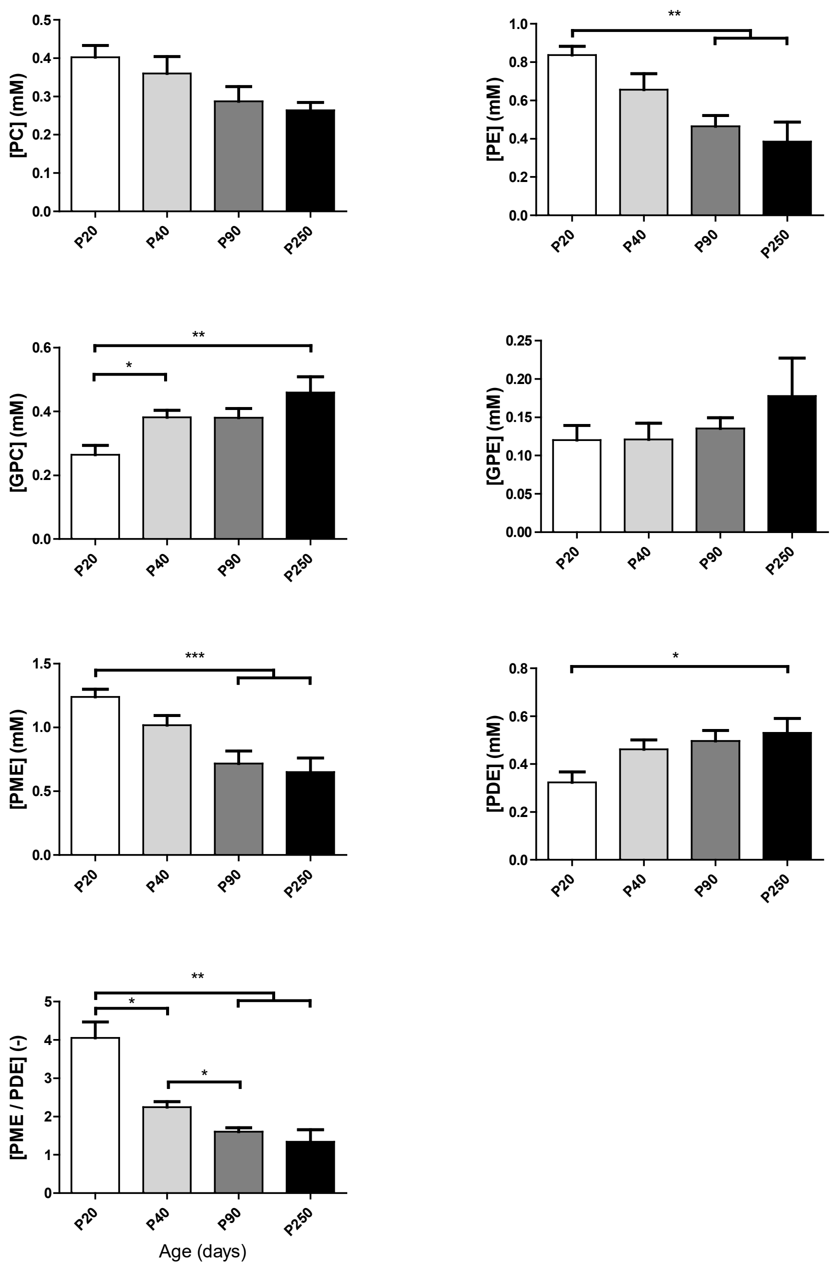

Figure S1: Changes of individual brain phosphoester compounds with age. Phosphomonoester (PME) is the sum of PC + PE, and phosphodiester (PDE) is the sum of GPC + GPE. The change of PME/PDE with age reflects decreased membrane turnover from postnatal day P20 to P250. All values are the mean ± SEM and significant differences are derived from the post hoc Bonferroni correction test for multiple comparisons. $**P < 0.01$, $****P < 0.0001$

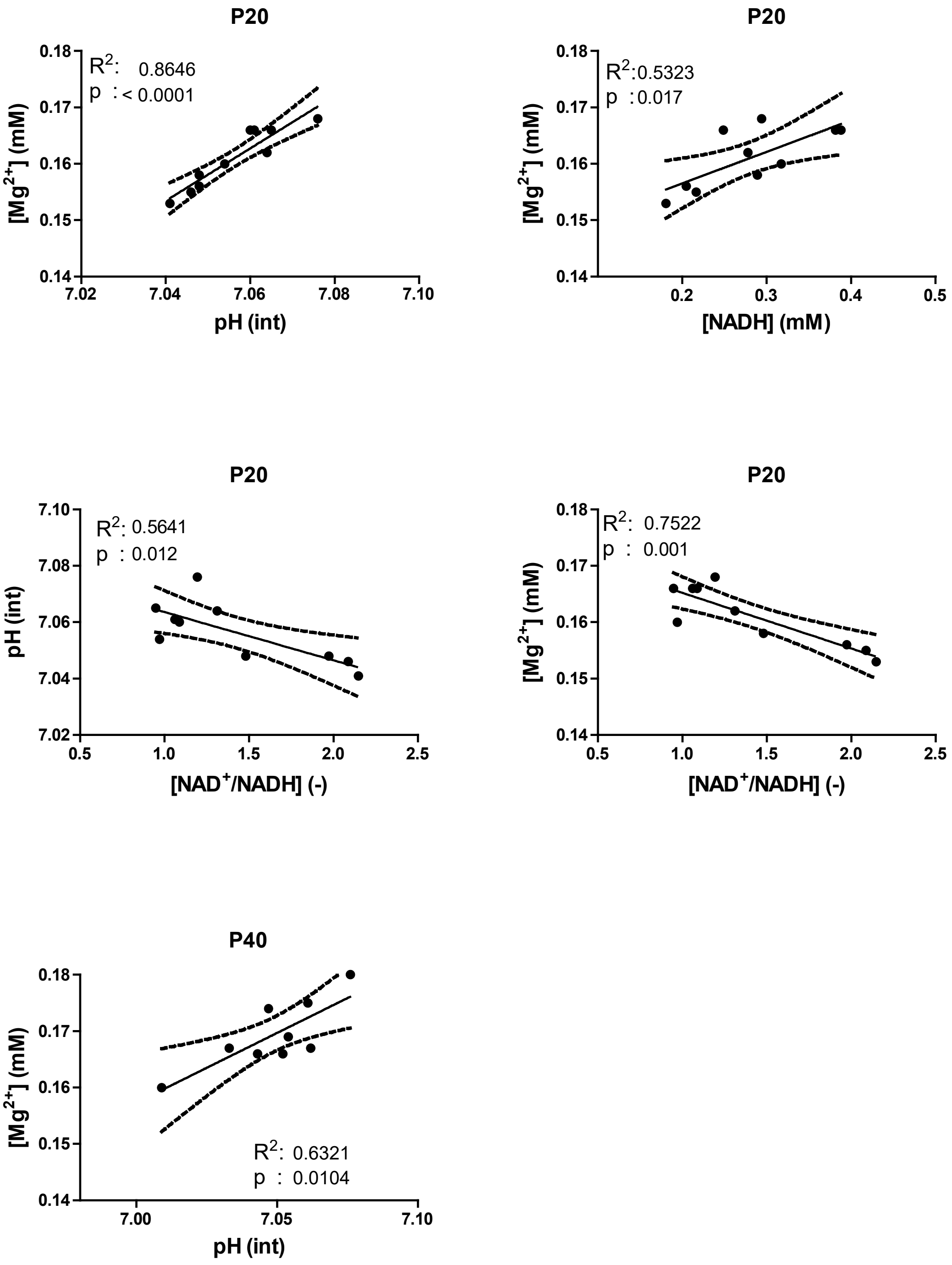

Figure S2: Plots of significant correlations between pH^{int}, free $[Mg^{2+}]$ and NAD metabolites. P values are depicted together with correlation coefficients R^2 obtained from the two-tailed Pearson correlation.

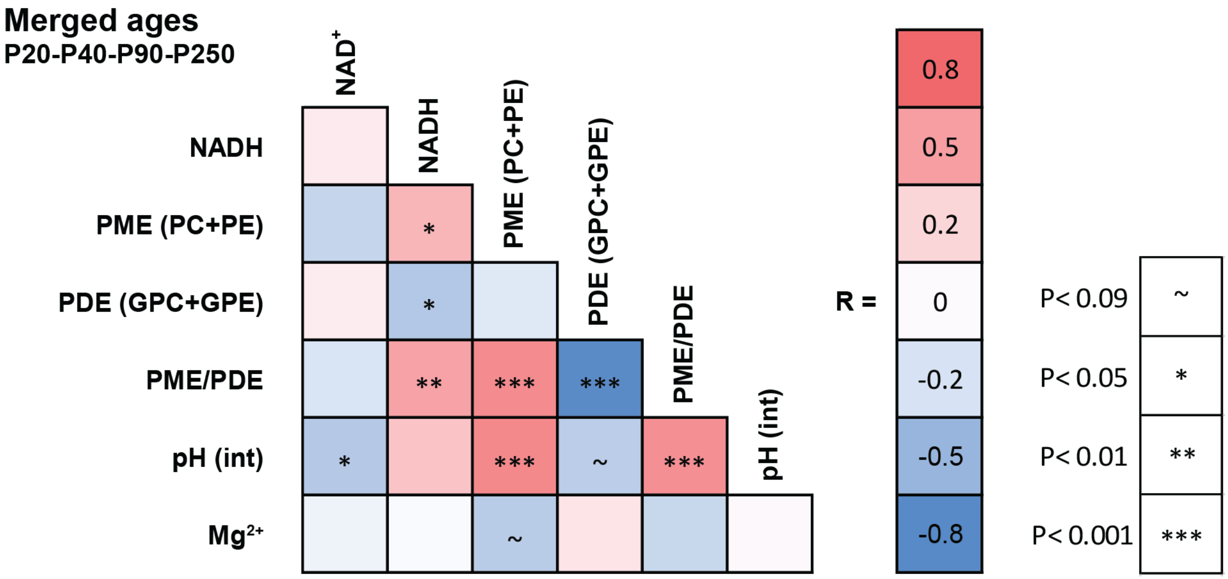

Figure S3: Correlation heat map of NAD^{+}, NADH, phosphoester, pH^{int} and free Mg^{2+} content in the mouse brain for merged data from all ages.
